# Supplementary material for: The polymorphism and tradition of funerary practices of medieval Turks in light of new findings from Tuva Republic
Source: PLoS One. 2022 Sep 22;17(9):e0274537. doi: 10.1371/journal.pone.0274537 (PMC9499277; doi:10.1371/journal.pone.0274537)
Supplement: Attachment — Submitted filename: review 280422.docx [file pone.0274537.s001.docx]

This is an interesting study and the authors have collected new archaeological evidence of Turkic funerary customs and, more generally Turkic cultural heritage in Central Asia. The paper is well written and structured. The manuscript comes as a result of promising field research and excavations in the Valley of the Kings in Tuva. The article provides an exhaustive list of literature, taking into account the latest research. The paper can be relevant to a broader readership, such as archaeologists, cultural anthropologists, ethnographers, and historians. The archaeological context is well presented for Central Asia in the Early Middle Ages. The authors discuss a broad range of cultural analogies published by other authors. The originality and interpretative ideas of the authors are based on new radiocarbon data, which confirms the legitimacy of correlating described monuments with the time of the Turkic Khaganate and the era of the early Middle Ages.

However, in my opinion, the paper has some shortcomings in regards to some formal aspects and text, and I feel that collected evidence has not been utilized to its full extent. Below the authors and the editor can find the remarks.

-line 555-556: while discussing the meaning of fences in medieval burial practices of the region, I strongly support the idea of the memorial purpose of fences – the so-called “ogradki”; since there are no arguments in favor of the fact that these are cenotaphs, and I agree with the arguments of the authors against the fact that these are cenotaphs.

- Figs 5, 8: In the article, we do not see an analysis of the typology of the bits and stirrups found on the monument. Also, the latest research on this problem is not cited (Seregin 2017; 2018).

-When analyzing the culture of the early Middle Ages, it would be useful to cite the work of Dosymbaeva (Dosymbaeva 2006).

-line 665: The problem is that we cannot track the exact origin of the Turkic as an ethnic group. The information/assumption that the Turks originated from the Altai has not yet been confirmed in archaeological materials. There is no such phenomenon as the "culture of the Turks" in the vastness of central Eurasia. It can also be interpreted as many different cultures. Whether different cultures can be called polymorphism of "Turkic culture" is an open question. Consider in the discussion.

-line 434, Fig.11: It should be noted that the monuments studied by the authors are not analyzed from the point of view of the spatial orientation of the graves and bodies interred. It is known, that the Turks had certain preferences in this respect: an eastern alignment of sacred objects, burials, dwellings. But the article does not consider the question of the monument's compliance with this cultural trait. Culturally based preferences (esp. E-W alignment) typical for Turks are evident in Fig.11, for example. Consider updating the manuscript.

-lines 373-374: It would be useful to use Western examples of funerary "fences" - in the form of typologically similar ditches in the burial mounds of the Khazars (Kruglov 2018) and the fence of the Voznesensky complex on the Dnieper (Ambrose 1982). They are generated by the same Turkic cultural environment and reflect the same ideas as the "fences" in Central Asia. In the Voznesensky memorial complex, we see traces of post-funeral rituals, when burned stirrups and weapons were placed in a pit after regular post-funeral feasts for several years.

-Fig.1. Formal correction: could you consider adding the names of the countries on the map.

-Fig.3. Formal correction: could you consider adding the depths (picture B for example)? Some more technical information about these soil covers (type etc.) would be useful e.g. cracking caused by…, consider adding a citation to some soil studies if necessary.

-Fig. 4. Formal suggestion: illustration is showing Tunnug 1. Medieval artifacts. Iron horse bits- can you add some more information, what elements is it exactly, what material is it made of, state of preservation (partially/full preserved, etc.), museum collection number? The same applies to Fig. 4-6; very vague description, and it can be improved. This is rather an en masse material, but in Fig.7 there is some strange note on a picture ‘maybe not include’- please remove it, or correct it differently.

- Fig. 8- Bronze finds from Objects 6 and 7- can you describe or name these items? function etc. Perhaps it could be a good idea to provide in descriptions some information about typologies of arrowheads by citation of appropriate published works. Fig. 8- scale in cm. Unify the fonts and scales in all illustrations. In Figs. 7-8 treat every item separately labeling them with numbers or letters, arrowhead 1, 2,3 – feature number 6, arrowhead 4,5,6,7 -feature number 27, etc. Fig. 9- maybe adding the author of the photo: photo by XY, that applies to all photos and drawings in the manuscript, drawn by/photo by, or if taken from a book, drawing after citation;

-Fig.14: formal suggestion: in the description please add dimensions of the stele, the stone material it was made of, and optionally weight. It would be useful to mark pictures a,b,c; the stele had been decorated, and there is some almond shape in the upper part, can you comment on that? is this perhaps anthropomorphic, or if not, add some comment regarding potential decorations. Add the author of these photos.

-line 575,587-590- change of the font? there is something wrong with the font format here; please also check the citation format: example: Linghu 1971: 910 (vol. 50, biography no. 42, “Tujue”, or Wang 1935: 1784 (vol. 100); these are Chinese works but the format of citation should match modern standards somehow; the dating of Chinese Dynasties if mentioned also should have AD/BC.

-Fig.15 I would like to suggest inserting item no. 4 in a horizontal position so the illustration keeps a more regular shape.

-lines 613-614 ‘Russian archaeologists such as V.V. Gorbunov, G.V. Kubarev, V.D. Kubarev, N.N. Seregin, A.A.Tishkin, have published compendiums of these artifacts… etc. Please add all mentioned works as citations by the end of the sentence.

But, despite these remarks, the article can be published without any doubt. The authors' conclusions are based on a large amount of factual material and correct theoretical generalizations. Below I recommend some more works which the authors may find useful for their manuscript in revison process:

Ambrose. A.K. About the Voznesensky complex of the VIII century on the Dnieper — a question of interpretation // Antiquities of the epoch of the Great Migration of peoples V to VIII centuries. Moscow: 1982. pp. 204-222. In Russian

Dosymbaeva A.M. Western Turkic Khaganate. Cultural heritage of the Kazakh steppe. Almaty, 2006. In Russian

Kruglov, E. V. FORMATION OF THE SOURCE BASE OF THE SOKOLOVSKY BALKATIP MONUMENTS // Nizhnevolzhsky Archaeological Bulletin. – 2018. – Vol. 17. – No. 1. – pp. 144-159. – DOI 10.15688/nav.jvolsu.2018.1.7. In Russian

Seregin, N. N. Stirrups from the burial complexes of the early medieval Turks in Mongolia // Peoples and religions of Eurasia. – 2017. – № 3-4(12-13). – Pp. 9-23.

Seregin, N. N. Horse bits and cheeks from early medieval Turkic burials in Mongolia // Izvestiya Altaiskogo gosudarstvennogo universiteta. – 2018. – № 2(100). – Pp. 174-181. – DOI 10.14258/izvasu(2018)2-29. In Russian

In Russian:

Серегин, Н. Н. Стремена из погребальных комплексов раннесредневековых тюрок Монголии // Народы и религии Евразии. – 2017. – № 3-4(12-13). – С. 9-23.

Серегин, Н. Н. Удила и псалии из погребальных комплексов раннесредневековых тюрок Монголии // Известия Алтайского государственного университета. – 2018. – № 2(100). – С. 174-181. – DOI 10.14258/izvasu(2018)2-29.

Амброз А.К. О Вознесенском комплексе VIII в. на Днепре — вопрос интерпретации // Древности эпохи великого переселения народов V-VIII вв. М.: 1982. С. 204-222.

Досымбаева А.М. Западный Тюркский каганат. Культурное наследие казахской степи. Алматы, 2006.

Круглов, Е. В. Формирование источниковой базы памятников типа Соколовской балки. Шилов В.П.: курганный могильник Ордынский бугор // Нижневолжский археологический вестник. – 2018. – Т. 17. – № 1. – С. 144-159. – DOI 10.15688/nav.jvolsu.2018.1.7.
