# Supplementary material for: The polymorphism and tradition of funerary practices of medieval Turks in light of new findings from Tuva Republic
Source: PLoS One. 2022 Sep 22;17(9):e0274537. doi: 10.1371/journal.pone.0274537 (PMC9499277; doi:10.1371/journal.pone.0274537)
Supplement: Attachment — Submitted filename: Response to Reviewers.docx [file pone.0274537.s002.docx]

15.06.2022

Dr. Mario Novak

Academic Editor

PLOS ONE

Dear Editor,

We are submitting with this rebuttal letter our revised manuscript “**The polymorphism and tradition of funerary practices of medieval Turks of Central Asia in light of new findings from Tuva Republic**” (slight change of wording in title) for your consideration. Please see enclosed documents. We list below changes that have been made in response to the reviewers’ concerns and suggestions and the additional references considered.

**18.07.2022 Addition:**

The second resubmission was sent back by the manuscript check. We address the raised points below:

*1. It is important that you include a cover letter with your manuscript. Please ensure that this letter is addressed specifically to PLoS ONE. Please also include*

** why this manuscript is suitable for publication in PLoS ONE.*

** how does your paper provide a worthwhile addition to the scientific literature?*

** how does your paper relate to previously published work?*

** which types of scientists do you believe will be most interested in your study?*

I REPEAT: We had included a cover letter – as always. It was addressed to PLOS ONE as can be seen above. As this is a resubmission of a minor revision, we believe that the editor has already judged the manuscript being potentially suitable for and interesting to the audience of PLOS ONE.

For the manuscript checker’s convenience we add the statement again:

In this work, we contribute new data and an interpretative framework for the variability of Turkic funerary rituals in the Eastern Steppe. The medieval Turks of the eastern Asian steppe are known for funerary finds exalting horsemanship and military heroism that thrived on intertribal warfare. Existing bodies of research on various categories of objects — which include architecture, stelae, grave goods and inhumations — are in depth but highly regionalized. As a result, our understanding of the archaeological culture of the Turks on a spatio-temporal scale commensurate with territorial shifts in their political diminion from the First Turkic Khanate through the Second Turkic Khanate (mid-6th to mid-8th centuries) remains disjunct. This paper addresses this problem of disparate data. We present a synthesis of Turkic archaeological research spanning Mongolia, southern Siberia, and Xinjiang in view of results of the excavation of medieval burials at Tunnug 1 in Tuva Republic— where Turkic remains are dispersed and not easily distinguishable from other funerary cultures of connecting time periods. We argue that Turkic funerary culture can be better characterized as polymorphic, which describes the presence of different regional amalgams of burial traditions. The horse-and-human burials and commemorative ogradka known to be quintessentially Turkic are but one of the more dominant amalgams. This pattern of differential practices is congruent with the history of the Turks as peoples of different lineages and political groupings, rather than people of a unitary culture.

*2. Please upload a Response to Reviewers letter which should include a point by point response to each of the points made by the Editor and / or Reviewers. (This should be uploaded as a 'Response to Reviewers' file type.)*

As in the previous submission you can find the Response to Reviewers point by point below.

*3. Thank you for including your ethics statement on the online submission form: "Field research was conducted under licenses No. 0434–2018 and No. 0590–2019 issued to T.S. from the Institute for the History of Material Culture, Russian Academy of Sciences. The permits were issued by the Russian Ministry of Culture." To help ensure that the wording of your manuscript is suitable for publication, would you please also add this statement at the beginning of the Methods section of your manuscript file.*

We added the statement to the methods section. Thank you.

*4. We note that you have referenced (Kilunovskaya et al. 2015, Kilunovskaya et al. 2017,Sadykov 2017b, Mongush and Mongush 2020) which has currently not yet been accepted for publication. Please remove this from your References and amend this to state in the body of your manuscript: (ie “Bewick et al. [Unpublished]”) as detailed online in our guide for authors*

I REPEAT: This has been noted before and it is likely a problem with an automated check that does not read the citations correctly. All sources have been published.

*5. Thank you for your response regarding the potential copyright of your Figure. We apologize for any delays incurred as a result of our checks for copyright.*

*Regarding Figure 1, please respond to the following:*

*a) As you note you have used ArcMap for the creation of this figure, please update your Figure caption to include attribution to ESRI. Upon resubmission, this should be good to proceed.*

Added ESRI attribution.

**07.06.2022 Addition:**

As the first resubmission was rejected by the manuscript check, we address the raised points below:

1. We note that the grant information you provided in the ‘Funding Information’ and ‘Financial Disclosure’ sections do not match. Please see below for your reference.

We updated the funding information to match the paragraph in the manuscript.

2. It is important that you include a cover letter with your manuscript. Please ensure that this letter is addressed specifically to PLoS ONE.

We had included a cover letter – as always. It was addressed to PLOS ONE as can be seen above. As this is a resubmission of a minor revision, we believe that the editor has already judged the manuscript being potentially suitable for and interesting to the audience of PLOS ONE.

3. We note that Figure 1 in your submission contain [map/satellite] images which may be copyrighted. All PLOS content is published under the Creative Commons Attribution License (CC BY 4.0), which means that the manuscript, images, and Supporting Information files will be freely available online, and any third party is permitted to access, download, copy, distribute, and use these materials in any way, even commercially, with proper attribution. For these reasons, we cannot publish previously copyrighted maps or satellite images created using proprietary data, such as Google software (Google Maps, Street View, and Earth). For more information, see our copyright guidelines: <http://journals.plos.org/plosone/s/licenses-and-copyright>.

We only use open source satellite data and have specified this in the captions of the figures as well as acknowledged the sources. @manuscript checkers: PLEASE, read this! This is by far the most annoying part about submitting at PLOS ONE and has delayed previous publications of the authors by months (and we are not the only ones who are held back by it – we generally know this to be a problem for colleagues who use open source satellite data).

4. Please include your full ethics statement in the ‘Methods’ section of your manuscript file. In your statement, please include the full name of the IRB or ethics committee who approved or waived your study, as well as whether or not you obtained informed written or verbal consent. If consent was waived for your study, please include this information in your statement as well.

We have obtained field permits and listed them. There is no additional ethics committee other than the state issuing permits. We do not work on human subjects i.e. no written or verbal consent possible. This doesn’t make sense. Thank you.

5. We note that you have referenced (Kilunovskaya et al. 2015, Kilunovskaya et al. 2017,Sadykov 2017b, Mongush and Mongush 2020) which has currently not yet been accepted for publication. Please remove this from your References and amend this to state in the body of your manuscript: (ie “Bewick et al. [Unpublished]”) as detailed online in our guide for authors

This has been noted before and it is likely a problem with an automated check that does not read the citations correctly. All sources have been published.

6. Please ensure that you refer to Figure 2 in your text as, if accepted, production will need this reference to link the reader to the figure.

This one actually makes sense. We put in a reference to Figure 2. Thank you.

**15.06.2022 Original Resubmission**

**Funding Statement**

We would like the funding statement to read as follows:

“The funders had no role in study design, data collection and analysis, decision to publish, or preparation of the manuscript. The excavation project is conducted with the financial and logistical help of the Russian Geographical Society (N 53/04/2019) and financial support of Dr. F. Paulsen, the Society for the Exploration of EurAsia, and the Russian Ministry of Culture (project No. 656-01-1-41/12-18). Post-excavation works of T. S. and J. B. were carried out within the framework of the Programs of Fundamental Scientific Research of the Russian Academy of Sciences, State Assignments No. 0160-2020-0002 and 0184-2019-0011 respectively. G. C. was funded by the Swiss National Science Foundation, grant number P400PG_190982.”

**MAIN TEXT**

- [from Reviewer 2] Reviewer 2 advised examining the proposed “polymorphism of ‘Turkic culture’” against the validity of the concept of the “culture of the Turks”. We agree with Reviewer 2 that the ethnic origin of the medieval Turks cannot be ascertained as their lineages are varied and ill-defined in historical literature. The “polymorphism” in our hypothesis does not refer to the Turks’ ethnic identity, however. It describes solely the nature of the archaeological record in question. In using “polymorphism”, we are not making a claim about the ethnic composition of the Turks, rather, we are suggesting that the “archaeological culture” (i.e. the pattern of material traits) of the Turks can be better made sense of if we were to consider the elusive and mixed lineages of certain Turkic tribes in medieval history, i.e. precisely what Reviewer 2 thought we might have overlooked, instead of as a discrete, homogenous cultural landscape.

Since our claim does not contradict the concern raised by Reviewer 2, no changes to the text are made on this point.

- [from Reviewer 1] We disagree with Reviewer 1’s suggestion to use “horsemanship” instead of “mounted combat”. The former denotes simply the practice of riding on horseback, the latter includes the element of fighting. The preponderance of weaponry in the Turkic burial tradition in question points to the latter.
- [from Reviewer 1] We ensured that the same terms are used throughout: “khaganate”; “Gokturks” “Turkic”, “Altai”, and “structure”. In some instances, we have replaced the term “khaganate” with “empire” as it better characterizes the nature of the political and tribal unions in the respective time periods.
- [from Reviewer 1] we addressed the confusion of the description “gold and metal jewelry” (line 150) by changing it to simply “jewelry”.
- [from Reviewer 1] We removed the species-specific comparison of the dog remains. The statement now reads “[The bones] belong to a medium-sized dog that stood around 40 cm tall and weighed 15-20 kg fully grown.”
- [from Reviewer 2] Reviewer 2 suggested analyzing the spatial orientation of graves and bodies interred with reference to Turkic burial tradition but contrary to what the reviewer noted, this information has been already been provided in the submitted manuscript in the first paragraph of sections “Structural types and heritage” and “Types of interment and subterranean structural layout”. As we described, the preference for the east-west orientation is only shown in Structure 45 at Tunnug (fig. 11). We now added that in the broader region of Tuva Republic, the most common direction of interment for human with horse burials is human head to the east and horse head to the west.
- [from Reviewer 2] We added CE to dynastic dates provided in section “ Types of interment and subterranean structural layout”, in the statements “the reigns of Western Wei and Northern Zhou (535-557 CE; 557-581 CE), and of Sui dynasty (581-618 CE),” “early in the 7th century CE, as described by a passage in *The New Book of Tang* (Xin Tang-shu 新唐書) recorded in 628 CE”, as well as in the statement “the khaganate established by the Gokturks (552-603 CE)...” in the introduction of the paper.
- [from Reviewer 2] Following Reviewer 2’s reminder, we added the respective references to the statement “Russian archaeologists such as V.V. Gorbunov, G.V. Kubarev…” under “Discussion in the section “Burial objects” as well as the “Subsequent field-defining studies include books (Klyashtorny, Savinov 2005; Kubarev 2005), as well as to the statement “numerous books and articles by V.V. Gorbunov, G.V. Kubarev, V.D. Kubarev,...” in the section “Altai and the Minusinsk Basin”.
- [from Reviewer 1] Reviewer 1 noted that a recent publication by G.V. Kubarev put forward a hypothesis of attributing medieval “Turkic” burials in the Russian Altai to the Karluks which were purportedly an autochthonous tribe. Since identifying the “ethnicity/ies” or Turkic subgroups responsible for the archetypal Turkic burials with a horse is not the principal concern of our paper, we consider Kubarev’s novel but conjectural theory, which is built on a two-part analysis of tamga signs and historiography, to be tangential to our research question. We therefore do not include it in our discussion.

**FIGURES**

All figures [Reviewer 2] Add author of all photos, drawn by, etc.

FIG. 1 [Question from Editor]

We properly acknowledged the image sources and creators of photos and maps. Added acknowledgement of SRTM DEM (public domain).

[Reviewer 1] add geographical landmarks

[Reviewer 2] add country names

We added country borders and names for orientation purposes.

FIG. 3 Add depth and soil type

FIGS. 4-6 [Reviewer 2] add information about the artifacts, material, state of preservation, museum collection number

FIG. 7 [Reviewers 1 and 2] Explain “ob. 27 may not be included”

FIG. 8 [Reviewer 1] Check scale of plaques and buckles

[Reviewer 2] suggest adding analysis of the typology of bits and stirrups and cite Seregin (2017, 2018). Relabel items with numbers and letters

FIG. 13 [Reviewer 1] Explain triangles. Add to caption.

FIG. 14 [Reviewer 2] Add dimensions of stele, stone material, weight, describe carved decoration.

FIG. 15 [Reviewer 2] Insert no. 4 in horizontal position

TABLE 1. what software was used to calibrate radiocarbon dates?

[Reviewer 1] Add map with location of Tunnug

We highlighted Tunnug 1 in the overview map (Fig. 1). Added Tunnug 1 to Figure 1 (red diamond).

**CHANGES TO REFERENCE LIST:**

- We verified that Kilunovskaya et al. 2015, Kilunovskaya et al. 2017, Sadykov 2017b, Mongush and Mongush 2020 have been published.
- Dmitry Savinov’s (2013) publication title is given in both Cyrillic and Latin transliteration, in the same style as all other references in Russian.
- [Reviewer 2] Suggest citing Dosymbaeva 2006
- [from Reviewer 2] We have checked that the font is showing up correctly – in Times New Roman – for the references of Linghu 1971 and Wang 1935 in the bibliography. We formatted in-text citations according to standard citation styles.

We added the following references recommended by Reviewer 2 and integrated the collections discussed in both these papers into our discussion (under “Burial objects”).

Seregin, N. N. Stirrups from the burial complexes of the early medieval Turks in Mongolia // Peoples and religions of Eurasia. – 2017. – № 3-4(12-13). – Pp. 9-23.

Seregin, N. N. Horse bits and cheeks from early medieval Turkic burials in Mongolia // Izvestiya Altaiskogo gosudarstvennogo universiteta. – 2018. – № 2(100). – Pp. 174-181. – DOI 10.14258/izvasu (2018): 2-29.

The following references, also recommended by Reviewer 2, however, are not added for reasons detailed below:

Ambrose. A.K. About the Voznesensky complex of the VIII century on the Dnieper — a question of interpretation // Antiquities of the epoch of the Great Migration of peoples V to VIII centuries. Moscow: 1982. pp. 204-222. In Russian

– Reviewer 2 suggested comparing our data to funerary fences in Khazar burial mounds in the Voznesensky complex but the focus of the remains considered in this paper is geographically and contextually too far removed to be considered a viable comparative archaeological assemblage for the purpose of explaining Turk activities in Inner Asia. Ambrose’s paper is concerned with remains of another Turkic people in the Dnieper region (present day Ukraine).

Kruglov, E. V. FORMATION OF THE SOURCE BASE OF THE SOKOLOVSKY BALKATIP MONUMENTS // Nizhnevolzhsky Archaeological Bulletin. – 2018. – Vol. 17. – No. 1. – pp. 144-159. – DOI 10.15688/nav.jvolsu.2018.1.7. In Russian.

— Same reason as above. Kruglov’s paper is concerned with Turkic remains in the Pontic Steppes, too distant from our research area and question to allow for a substantive comparison.

Dosymbaeva A.M. Western Turkic Khaganate. Cultural heritage of the Kazakh steppe. Almaty, 2006. In Russian

– this is a big and interesting monograph that focuses to the “sanctuaries” of the Western Khaganate and the possible cultural differences between the sites of the Western and the Eastern Khaganates. This is an important study, but too far from our topic.

We thank the reviewers for their thorough review of our manuscript and your helpful comments. We look forward to receiving your decision on our revised submission.

Sincerely,

Annie Chan, Timur Sadykov, Jegor Blochin, Irka Hajdas, Gino Caspari
