# Supplementary material for: The polymorphism and tradition of funerary practices of medieval Turks in light of new findings from Tuva Republic
Source: PLoS One. 2022 Sep 22;17(9):e0274537. doi: 10.1371/journal.pone.0274537 (PMC9499277; doi:10.1371/journal.pone.0274537)
Supplement: Attachment — Submitted filename: We addressed the following comments.docx [file pone.0274537.s003.docx]

We addressed the following comments:

Reviewer #1: I believe that the revised article can be published. Please correct the typo: page 4 line 2: Seregin and Matrenin (not Maternin)

Typo corrected.

Reviewer #2: All comments have been addressed

Thank you.
